# Strengthening malaria and climate research in Ethiopia

**DOI:** 10.1186/1475-2875-13-S1-P56

**Published:** 2014-09-22

**Authors:** Bernt Lindtjørn, Eskindir Loha, Wakgari Deressa, Meshesha Balkew, Teshome Gebremichael, Asgeir Sorteberg, Adugna Woyessa, Abebe Animut, Korecha Diriba, Fekadu Massebo, Ellen Viste, Torleif Markussen Lunde, Dereje Tesfahun

**Affiliations:** 1 University of Bergen, Bergen, Norway; 2 Addis Ababa University, Addis Ababa, Ethiopia; 3 Arba Minch University, Arba Minch, Ethiopia; 4 Hawassa University, Hawassa, Ethiopia; 5 National Meteorology Agency, Addis Ababa, Ethiopia

## 

The project “Ethiopian Malaria Prediction System” implemented from 2007 to 2012 combined new population-based malaria transmission information with climate and land use variability data to develop an early warning tool to predict malaria epidemics in Ethiopia. Scientists from Ethiopia and Norway collaborated to incorporate climate variability and forecast information for malaria epidemics.

Our study shows that the association between weather and malaria is complex. Statistical models can predict malaria for large areas. However, as malaria transmission varies and depends on local environmental conditions, we need to have good and local knowledge about each area. However, weather variability is the main driver of malaria in Ethiopia.

While the generation of precipitation depends on local ascent and cooling of the air, our research provided new data on the transport of moisture into the country that may improve weather forecasting. We developed a new classification of climate zones, have mapped drought episodes in Ethiopia during the last decades, and have improved seasonal weather forecasting. Our hydrology studies show that potential climate change differs among the Ethiopian river basins, with river flows being sensitive to variations in rainfall, and less to temperature changes.

The computer model, Open Malaria Warning, incorporates hydrological, meteorological, mosquito-breeding, land-use data, and cattle densities to find out when and where outbreaks are likely to occur. We validated the model with data for malaria transmission in the highlands and lowlands, characterizing malaria transmission over some years in both highlands and lowlands. This provided us with new knowledge on malaria transmission in Ethiopia, how intense the seasonal transmission is, and how malaria occurs in different populations and areas. Our study showed that indigenous malaria transmission during a non-epidemic year takes place above 2000 m altitude. We also showed the ideal temperature for malaria transmission is about 25°C, underlining that global warming may lead to increased risk of malaria in highland areas, and less in the lowlands with already high average temperatures. However, to validate such models, there is a need for several years of active monitoring of malaria cases and mosquito densities. Unfortunately, such data is rare in Africa, and we need to invest in long-term monitoring of malaria transmission.

